# Melanoma complicating treatment with natalizumab for multiple sclerosis: A report from the Southern Network on Adverse Reactions (SONAR)

**DOI:** 10.1002/cam4.1098

**Published:** 2017-06-20

**Authors:** Rachel A. Sabol, Virginia Noxon, Oliver Sartor, Joseph R. Berger, Zaina Qureshi, Dennis W. Raisch, LeAnn B. Norris, Paul R. Yarnold, Peter Georgantopoulos, William J. Hrushesky, Laura Bobolts, Paul Ray, Akida Lebby, Robert C. Kane, Charles L. Bennett

**Affiliations:** ^1^ Tulane University School of Medicine New Orleans Louisiana; ^2^ The Southern Network on Adverse Reactions (SONAR) program University of South Carolina College of Pharmacy Columbia South Carolina; ^3^ Department of Neurology Perelman School of Medicine Philadelphia Pennsylvania; ^4^ The Arnold School of Public Health University of South Carolina Columbia South Carolina; ^5^ University of New Mexico College of Pharmacy Albuquerque New Mexico; ^6^ Oncology Analytics Plantation Florida; ^7^ The Medical University of South Carolina Hollings Cancer Center Charleston South Carolina; ^8^ William Jennings Bryan Dorn Veterans Administration Medical Center Columbia South Carolina

**Keywords:** Melanoma, multiple sclerosis, natalizumab

## Abstract

A 43‐year‐old female with multiple sclerosis developed urethral melanoma. The only potential risk factor was treatment with natalizumab, a humanized monoclonal antibody against *α*4 integrins. To investigate the risk‐exposure relationship, we reviewed this case, all other published cases, and cases of natalizumab‐associated melanoma reported to regulatory agencies. Data sources included the Food and Drug Administration's (FDA) Adverse Event Reporting System (FAERS) (2004–2014), a FDA Advisory Committee Meeting Report, and peer‐reviewed publications. In the United States, the manufacturer maintains an FDA‐mandated Tysabri Safety Surveillance Program (part of the Tysabri Outcomes Unified Commitment to Health (TOUCH)) of natalizumab‐treated patients. We statistically compared reporting completeness for natalizumab‐associated melanoma cases in FAERs for which information was obtained entirely from the TOUCH program versus cases where FAERS information was supplemented by TOUCH program information. FAERS included 137 natalizumab‐associated melanoma reports in patients with multiple sclerosis. Median age at melanoma diagnosis was 45 years (range: 21–74 years). Changes in preexisting nevi occurred in 16%, history of cutaneous nevi occurred in 22%, diagnosis within 2 years of beginning natalizumab occurred in 34%, and 74% had primary surgical treatment. Among seven natalizumab‐treated MS patients who developed biopsy‐confirmed melanoma on treatment and reported in the literature, median age at diagnosis was 41 years (range: 38–48 years); and the melanoma diagnosis occurred following a median of 12 natalizumab doses (range: 1–77 doses). A history of mole or nevi was noted in four patients and a history of prior melanoma was noted in one patient. Completeness scores for reports were significantly lower for FAERS cases reported from the TOUCH program versus FAERS cases supplemented by TOUCH information (median score of 2 vs. 4 items out of 8‐possible items, *P* < 0.0007). Clinicians should monitor existing nevi and maintain suspicion for melanoma developing in natalizumab‐treated patients. The TOUCH Safety Surveillance Program, currently focused on progressive multifocal leukoencephalopathy, should be expanded to include information on other serious complications including malignancies, particularly if they are immunologic in nature.

## Introduction

Natalizumab (Tysabri) is a humanized monoclonal antibody approved internationally for treatment of some forms of multiple sclerosis (MS). Natalizumab selectively blocks *α*4 integrins on the surface of lymphocytes, thereby preventing their adhesion to vascular‐cell adhesion molecule 1 (VCAM‐1). VCAM‐1 is expressed on surfaces of vascular endothelial cells in brain and spinal cord blood vessels 1. Blocking *α*4 integrins reduces further brain inflammation¸ apparently accounting for the drug's effectiveness for MS 2, 3, 4, 5. Natalizumab also inhibits expression of alpha4 integrin, which has been shown to prevent melanoma metastasis but also may promote melanoma invasion 3. The most serious complication of natalizumab is progressive multifocal leukoencephalopathy (PML), an adverse drug reaction (ADR) confirmed to have been reported in 714 patients through March 6, 2017, and a risk estimate of 4.2/1000 patients (95% confidence interval, 3.89‐4.52).^5^ PML is also a known complication of therapy with rituximab, an ADR initially reported in 2009.^6^ In 2012, receiving an immune suppressing medication prior to initiating natalizumab was identified as an additional risk factor for PML. Natalizumab‐treated multiple sclerosis persons with all three known risk factors (presence of anti‐JC virus antibdodies, longer duration of natalizumab treatment (specially beyond 2 years)), and prior treatment with an immunosuppressant medication) have an estimated risk of 11 per 1,000 users of natalizumab. 7, 8, 9, 10, 11.

The Southern Network on Adverse Reactions (SONAR) is a state/National Cancer Institute (NCI)‐funded pharmacovigilance program focusing on identifying rare but serious ADRs and complications involving drugs that are associated with immunologically related illnesses 12. In 2014, a SONAR collaborator treated a 43‐year‐old female who developed urethral melanoma shortly after natalizumab therapy was initiated. Following this safety signal, SONAR undertook a comprehensive investigation focusing on natalizumab‐associated melanoma.

## Material and Methods

### Data

Information on natalizumab and natalizumab‐associated melanoma were identified by reviewing the FDA’s Adverse Event Reporting System (FAERS) database (January 1, 2008‐ March 31, 2014), peer‐reviewed literature identified in PubMed [(2006‐ 2016)‐ MeSH terms: natalizumab and melanoma], a 2006 Natalizumab Advisory Committee Briefing Document prepared for the Peripheral and Central Nervous System Drugs Advisory Committee 2007 meeting, data obtained from the manufacturer on the numbers of patients and patient‐years exposure to natalizumab from November 23, 2004, to March February 28, 2017, the product label, and a March 2017 Tysabri‐Progressive Multifocal Leukoencephalopathy Updated Written Report from the manufacturer of natalizumab. 5, 6, 10, 14. Inclusion criteria included: use of natalizumab prior to melanoma diagnosis and diagnosis of melanoma based on pathology information included in the adverse event report (as in the seven case reports and the index case) or notations in FAERS reports that melanoma had been diagnosed (there was not communication back to the reporter seeking report verification).

Abstracted data included FDA's case identification number, source of each data item (FAERS report, the Tysabri Outreach Unified Commitment to Health (TOUCH) Safety Surveillance Program database, or other), patient characteristics (age, gender, and country from which the case was reported); treatment (length of time from first natalizumab dose to melanoma diagnosis, number of natalizumab doses received prior to melanoma diagnosis), outcome (vital status at last follow‐up); melanoma characteristics (site; preexisting nevi or other melanoma risk factors); association with the TOUCH Safety Surveillance Program database (for United States’ cases only); and information about the individual reporting the case (neurologist, other physician, nurse, pharmacist, patient, or family member). For the TOUCH Safety Surveillance Program database, clinical information on all natalizumab‐treated people in the United States is obtained initially at 3 months after treatment initiation and then at 6‐month intervals, and additional detailed information is obtained for all natalizumab‐treated persons who develop PML. The TOUCH Safety Surveillance Program is designed to facilitate early PML identification and subsequently to improve survival from PML with early treatment. No duplicate cases existed in the final sample.

### Classifying melanoma cases

Location of melanoma cases was categorized by site and by sun exposure. Site was classified as cutaneous, ocular, mucosal or unknown, and was determined from the primary site of melanoma indicated in individual case reports. For node‐rich regions, such as the axilla, the site of melanoma was characterized as cutaneous. Sun exposed and non‐sun exposed areas for cutaneous melanoma were determined using scales described previously 15, 16.

### Scoring system for evaluating reporting completeness in FAERS

As in prior work, completeness of reporting of individual cases was evaluated based on a system that focused on clinical and treatment‐related date elements 12. An 8‐point clinical information checklist was derived with input from collaborating clinicians. The 8 items included: site of melanoma, lymph node status, Breslow depth, preexisting nevi, family history of melanoma, prior immunosuppressive treatment, survival and start date of melanoma.

### Analysis

Descriptive statistics were used to determine the characteristics of the cases of natalizumab‐associated melanoma based on reporting source, (United States vs. ex‐United States) and from FAERS‐supplemented by data obtained from the TOUCH Safety Surveillance Program, FAERS data that was entirely obtained from the TOUCH Program, and FAERS data that had no data obtained from the TOUCH Program included in the report. Descriptive statistics were computed using SAS 9.4 (Cary, NC). Data were compared between United States’ versus non‐United States’ sources using exact nonparametric discriminant analysis 17.

## Results

### Individual case report—the signal for this investigation

A 43‐year‐old female patient with MS reported difficulty urinating for 1 month. The patient had been diagnosed with MS 6 years earlier, presenting with optic nerve involvement. The patient had received natalizumab treatment for 2 years and 2 months, after previous failure of interferon beta therapy of MS. She had no personal or family history of melanoma. Cystoscopy evaluation identified a polyp in the distal urethra measuring 1.0 × 0.7 × 0.5 centimeters. The patient was suspected to have urethral melanoma. Pathology confirmed the diagnosis of melanoma and the surgical biopsy margins were positive for melanoma. Dermatology evaluation did not identify other suspicious primary or metastatic areas. Natalizumab was discontinued. PET scan revealed invasion into the bladder but no evidence of distant metastases. Surgical resection included an anterior pelvic exenteration with cystectomy, partial vaginectomy, hysterectomy, and urethrectomy. Five months later, the patient reported increasing vaginal pain and bleeding with heavy green‐colored discharge. The patient was afebrile and no odors or other changes were noticeable. Physical examination demonstrated irritation and inflammation around the site of the urethra. A vaginal exam identified a mass on the left side wall, which was subsequently biopsied and found to be recurrent melanoma. The patient subsequently died from widely metastatic malignant urethral melanoma. The patient did not receive systemic antineoplastic therapy as per her choice.

### FAERs review

**Figure 1:**
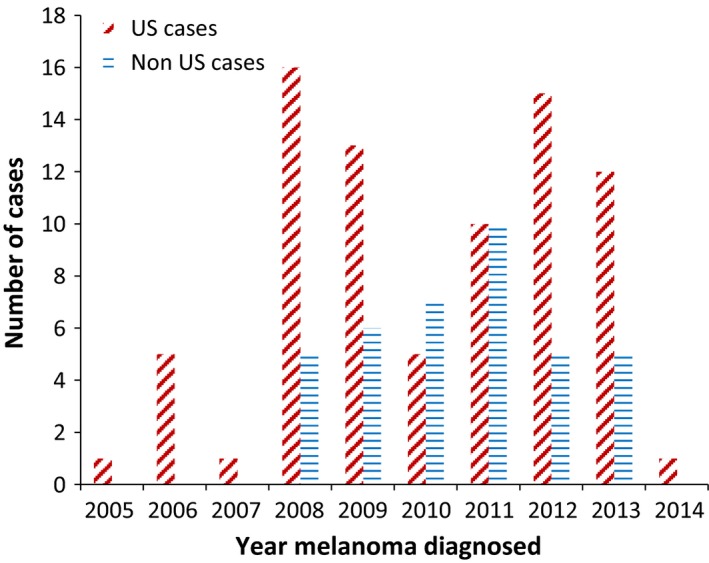
Year when natalizumab‐associated melanoma cases from the United States (US) and other countries (Non US cases) were reported to the Food and Drug Administration. Data source: FDA’s Adverse Event Reporting System (FAERS) database (January 1, 2008‐ March 31, 2014).

Of 141 cases of natalizumab‐associated melanoma included in FDA's FAERS reports prior to April 1, 2014, two cases each were duplicated, yielding an initial data set of 139 cases. (It should be noted that an additional 45 cases of natalizumab‐associated melanoma were reported between April 1, 2014 and June 30, 2016). Of the 139 cases, two did not list natalizumab as the drug of interest and were thus excluded. Most cases were reported between 2008 and 2013. (Fig. 1) All cases reported from countries other than the United States were reported between 2008 and 2013, while cases reported for the United States were reported between 2005 and 2014. (Fig. 1) Females accounted for 77% of cases (Table 1). All but two cases received natalizumab for a diagnosis of MS. Median patient age at diagnosis of melanoma was 45 years (range: 21–74 years). Of the nine cases who died from melanoma, seven were from the United States. The majority (74%) of melanoma cases were treated with surgery. Overall, 16% of the cases indicated a change in preexisting nevi and 22%, indicated a history of cutaneous nevi. The majority of melanoma cases were diagnosed within 2 years of beginning natalizumab therapy.

**Table 1:** Characteristics of FAERS patients who developed natalizumab‐associated melanoma (*n* = 137 cases)

	Total	US	Non‐US
FAERS	*N* = 137	*N* = 95 (69%)	*N *= 42 (31%)
Age (median [range])	45 [21, 74]	47 [21, 74]	39 [21, 63]
Number of updates reported to the FDA (median)	2	2	1
Number of months of information from the time of melanoma diagnosis (median)	4	5	4
Female (%)	106 (77)	73 (77)	33 (79)
Disease natalizumab prescribed for *N* (%)			
Multiple Sclerosis	135 (98)	93 (98)	42 (100)
Crohn's	1 (1)	1 (1)	0
Not Known	1 (1)	1 (1)	0
Melanoma Site *N* (%)			
Cutaneous	104 (76)	73 (77)	31 (74)
Mucosal	2 (1)	2 (2)	0
Ocular	5 (4)	3 (3)	2 (5)
Not known	26 (19)	17 (18)	9 (21)
Site sun exposed N (%)			
Yes	88 (64)	62 (65)	26 (62)
No	23 (17)	17 (18)	6 (14)
Not Known	26 (19)	16 (17)	10 (24)
Time on natalizumab until melanoma diagnosis *N* (%)			
0–24 months	47 (34)	36 (38)	11 (26)
25–48 months	26 (19)	13 (14)	13 (31)
49–72 months	10 (7)	4 (4)	6 (14)
73–96 months	1 (1)	1 (1)	0
Not specified	53 (39)	41 (43)	12 (29)
Alive at follow‐up *N* (%)	128 (93)	88 (93)	40 (95)
Concomitant drug use *N* (%)	51 (37)	30 (32)	21 (50)
Nevi history *N* (%)			
Yes	30 (22)	18 (19)	12 (29)
No	30 (22)	20 (21)	10 (24)
Unknown	77 (56)	57 (60)	20 (48)
Melanoma treatment *N* (%)			
Chemotherapy	2 (1)	2 (2)	0
Chemotherapy and radiation	1 (1)	1 (1)	0
Surgery	92 (67)	61 (64)	31 (74)
Radiation	1 (1)	0	1 (2)
Surgery combination	9 (7)	9 (9)	0
Other	3 (2)	2 (2)	1 (2)
No	1 (1)	0	1 (2)
Not applicable	17 (12)	12 (13)	5 (12)
Unknown	11 (8)	8 (8)	3 (7)
TOUCH *N* (%)			
Data obtained from TOUCH^a^	N/A	14 (15)	0
FAERS report supplemented by TOUCH data^b^	N/A	69 (73)	0
FAERS data without any TOUCH data^c^	N/A	12 (13)	42 (100)
Change in Nevi *N* (%)			
Yes	22 (16)	14 (15)	8 (19)
No	115 (84)	81 (85)	34 (81)
Reporter *N* (%)			
Neurologist	37 (27)	28 (29)	12 (29)
Unknown	25 (18)	9 (9)	14 (33)
Patient	22 (16)	19 (20)	0
Nurse	16 (12)	16 (17)	1 (2)
Physician	9 (7)	3 (3)	6 (14)
Family	4 (4)	5 (5)	0
Registered nurse	5(4)	5 (5)	0
Investigator	5 (4)	1 (1)	5 (12)
Physician assistant	2 (1)	2 (2)	0
ANSM	2 (1)	0	2 (5)
Health care professional	2 (1)	2 (2)	0
Consumer	1 (1)	1 (1)	0
Doctor	1 (1)	1 (1)	0
Manufacturer report	1 (1)	1 (1)	0
Other authority	1 (1)	0	1 (2)
Assistant	2 (1)	1 (1)	0
Nurse practitioner	1 (1)	1 (1)	0

Case information was obtained from the FDA's Adverse Event Reporting System (FAERS). (Between April 1, 2014 and June 30, 2016, 45 additional natalizumab‐associated melanoma cases were reported to FAERS‐ 12 from ex‐United States countries and 33 from the United States. These cases are not included in Table 1).

a Cases reported to the FDA directly from the TOUCH Safety Surveillance Program.

b Cases reported to the FDA independent of TOUCH but with supplemental information obtained from the TOUCH Safety Surveillance Program.

c Cases reported to the FDA with no information obtained from the TOUCH Safety Surveillance Protocol.

Sources of FAERS reports were neurologists (29%), nurses (18%), patients (14%), family members (4%), physicians other than a neurologist (7%), or a clinician who was a study co‐investigator for a clinical trial with natalizumab (4%). No cases were reported by pharmacists, (although for all serious adverse events reported to the FDA, 90% of health care worker reported events are from pharmacists). Also, 20% of cases from the United States were reported by a patient or a family member versus 0% of cases from countries other than the United States. Overall, 17% of cases identified melanoma as having developed in areas of the body not considered to be sun exposed (Table 1). Individual case reports had a median of two updates of information sent to FAERS after the initial report had been submitted and a 5‐month time‐interval between the time of diagnosis of melanoma and initial submission of case information to the FDA or the drug manufacturer.

With respect to clinical information (e.g., site of melanoma), the median case reporting score was 4 items (maximum score of 8 items reported) (i.e., only 50% of clinically relevant items were included in adverse event reports). There was no statistically reliable difference in reporting completeness scores between cases reported from the United States versus other countries.

Among 95 FAERS cases of natalizumab‐associated melanoma reported from the United States, 15% were based on information entirely obtained from the TOUCH Program; 73% were cases where initial information was reported directly to FAERS and supplementary information obtained from the Registry was added; and 13% were reported to FAERS and no additional information from the TOUCH Program was added to the FAERS report (Table 1). There were significant differences in clinical completeness scores between cases where the TOUCH Program provided the entire case information to FAERS versus those cases where FAERs data were supplemented by TOUCH Program data (median score of 2 items vs. 4 items out of 8 possible items, *P* < 0.0007).

### Literature review

Case information for seven published cases was reviewed (Table 2) 2, 18, 19, 20, 21, 22, 23. Among the seven cases, six patients were female. The median age of the patients was 41 years at the time of diagnosis of melanoma (range: 38–48 years). Three patients were enrolled in clinical trials evaluating natalizumab when melanoma was diagnosed, including two patients who were enrolled on a prospective phase III randomized trial at the time of diagnosis of melanoma 18, 21, 24. One 38‐year‐old male was enrolled on the randomized phase III study comparing natalizumab versus placebo. This person received one dose of natalizumab prior to the diagnosis of recurrent melanoma 21, 24. One patient was a 48‐year‐old female, also on the same phase III clinical trial, who received 30 infusions of natalizumab on the clinical trial and was subsequently, in an open label phase of the trial, received five more infusions 21. Treatment was discontinued when the clinical trial was put on hold following reporting of three cases of progressive multifocal leukoencephalopathy 24. The third clinical trial participant was on a long‐term safety evaluation and was diagnosed with melanoma after receiving 77 doses of natalizumab 2. A history of mole or nevi was noted in four patients (including an ocular mole in one patient) and a prior history of melanoma in one patient. Melanoma was diagnosed after a median of 12 doses of natalizumab (range: 1–77 doses). Involved sites included an ocular melanoma (one patient), shoulder, arm, underside of a foot, and leg. Metastatic melanoma developed in one patient, and one patient died from melanoma.

**Table 2:** Clinical findings for seven published cases of natalizumab‐associated melanoma among persons with multiple sclerosis

Patient number, age, gender, family history	Clinical trial participant	# of doses	Clinical features	Other information
(1) 38, male, family history not reported^21^, ^23^	Clinical trial (AFFIRM study)^21^	Skin lesion noted at first dose; 5 doses when melanoma diagnosis was confirmed;	Patient died of melanoma	Died from melanoma
(2) 46, female, family history not reported 20	Nonclinical trial	1 dose when mole on shoulder started rapidly changing	Mole on shoulder for a long time prior to melanoma. Had metastatic spread to regional lymph nodes.	After discontinuing natalizumab, patient developed widely metastatic melanoma
(3) 39, female, MS, no family history 2	Nonclinical trial	5 doses—noted slow change in long‐standing mole on left leg	After 10 months ulcerated and ablated. Spreading melanoma, 1.6 mm thickness and Clark level IV. No metastasis to lymph nodes.	Therapy stopped after 16 doses,
(4) 41, female, France, no family history 22	Nonclinical trial	12 doses. Natalizumab continued after diagnosis for 21 additional doses with close monitoring	Superficial spreading melanoma from a dysplastic nevi, upper arm	Had many atypical moles for years. Melanoma occurrence in close temporal relation to natalizumab start.
(5) 45, female, family history not reported 20	Nonclinical trial	Several doses. Mole increased in size and depth and became pigmented.	Ocular melanoma. Long history of mole in posterior eye. Mole was unchanged for 6 years prior to natalizumab	Family history of melanoma.
(6) 38, female, no family history of atypical nevi 19	Clinical trial evaluating natalizumab/ beta‐interferon and then on a long‐term clinical trial	Diagnosed after 77 natalizumab doses. 35 natalizumab doses (with interferon beta) and then on long‐term extension study of natalizumab	Malignant melanoma. Clark level III and Breslow index 0.5 mm.	No evidence of metastasis
(7) 48, female No family history of melanoma or prior immunosuppressive therapy 18	Clinical trial (initially on the AFFIRM study) 21	30 doses (300 mg every 4 weeks) of Natalizumab and enrolled in open label of trial with an additional 5 doses.	5 months after last dose noticed increase in mole on right shin. Lesion was flat with dark center.	No evidence of metastasis or recurrence

AFFIRM, the Natalizumab Safety and Efficacy Trial in Relapsing Remitting Multiple Sclerosis.

## Discussion

We identified an association of natalizumab treatment with melanoma. In interpreting our findings, several factors, particularly epidemiology of melanoma, natalizumab‐associated progressive multifocal leukoencephalopathy (PML), various aspects of the TOUCH Risk Management (RiskMAP) Action Plan, and the Safety Surveillance Program in this RiskMAP (Table 3), potential pathophysiology, and an overview of pharmacovigilance efforts for opportunistic complications of medications, should be considered 6, 7, 8, 9, 10, 11, 13, 24, 25, 26, 27, 28, 29, 30, 31, 32, 33, 34, 35, 36, 37, 38, 39, 40, 41, 42, 43, 44, 45, 46, 47.

**Table 3:** Summary of a Proposed Upgrade Natalizumab Risk Minimization Action Plan (RiskMAP), TOUCH^™^‐ as it pertains to pharmacovigilance, PML, and melanoma

(1) Prescribing program 47	
(1.1) Pharmacy and infusion center requirements	°All pharmacy and infusion center staff members are trained in adverse event reporting protocols such as 15‐day reporting of PML infection or melanoma occurrence, any other opportunistic infection and/or death Prior to infusion, center staff must ensure the following requirements are met: °The infusion site will complete a Pre‐Infusion Patient Checklist and confirm clearance from a prescriber Patients will be given a revised Medication Guide that has additional information on cutaneous nevi and melanoma, as well as time to read it
(1.2) Prescriber requirements	TOUCH^™^‐registered prescribers must agree and adhere to the following requisites for registration: °Demonstrate capability in diagnosing and managing PML, nevi, and malignant melanoma and other opportunist infections or opportunistic malignancies, or have access to specialists with this ability for referral °Provide patients with a natalizumab Revised Medication Guide °Report any cases of PML, melanoma, or hospitalization/death due to PML, melanoma, and/or any other opportunistic infection or opportunistic malignancy to the manufacturer °Evaluate patient 3 months following first infusion, 6 months following the first infusion, and every 6 months henceforth barring discontinuation of treatment, including a skin review and evaluation of all nevi for changes. °Determine every 6 months whether each patient should continue natalizumab and has not had significant nevi or other skin lesion develop and fill out the Revised Patient Status Report and Reauthorization Questionnaire
(1.3) Patient requirements	TOUCH^™^‐registered patients must adhere to the following conditions before being accepted for registration: °Must be TOUCH^™^‐registered. °Must understand the potential risks and benefits of treatment, including the increased possibility of PML infection, potential for nevi growth, and potential for melanoma development. °Revised Medication Guide must be read. °Information about concurrent medicines and treatments taken must be provided at each infusion. °Must have a baseline dermatologic evaluation
(1.4) Education Program	Educational materials on the benefits and risks associated with natalizumab, increased risk of PML, potential risks of melanoma, and requirements of the TOUCH^™^ program will be provided to prescribers, infusion site staff, pharmacists, and patients by the manufacturer
(2) (2.1) Healthcare provider and patient educational materials	Educational Materials and forms include the following: °Patient Revised Medication Guide and Revised Package Insert (for patients and prescribers). °Revised Patient Status Report and Reauthorization Questionnaire (filled out every 6 months by prescribers) °Revised Dear Doctor and Dear Patient Letters which include information on nevi and melanoma
(3) Reporting	The manufacturer has implemented a reporting and collection system for safety information as follows: All spontaneous and solicited adverse event reports from any postmarketing source are reported A report of all confirmed PML cases, melanoma cases, or nevi that have substantially changed are to be sent to the FDA within 15 calendar days Reports of any other serious opportunistic infections, opportunistic malignancies, and/or death must be sent to the FDA within 15 calendar days
(4) TOUCH^™^ safety surveillance program	The manufacturer, through TOUCH^™^, will systematically follow and actively solicit information regarding the occurrence of PML melanoma, and other serious opportunistic infections or opportunistic malignancies on every natalizumab‐treated patient in the U.S. The various reporting mechanisms include: ° Thorough collection and assessment of preinfusion revised patient checklists and the revised prescriber/patient enrollment form ° Thorough serious adverse event reporting ° Thorough contact with prescribers every 6 months in the form of a Revised Patient Status Report and Reauthorization Questionnaire ° Attempt to find patients who discontinued natalizumab treatment and track them for 6 months 1 The manufacturer is creating a Safety Review Committee to review safety data and determine appropriate corrective actions, if needed
(5) TOUCH^™^ program evaluation	The manufacturer will evaluate the effectiveness of the natalizumab RiskMAP and will report results quarterly for the first year, then every 6 months for 2 years, and annually thereafter to FDA Each FDA submission will include analyses of two major datasets: °Health outcomes data (e.g., PML rate, melanoma rate, overall safety) Systems/process data, quality, and compliance metrics

In 2008, two patients with natalizumab‐associated melanoma were described in report from a Boston MS clinic. One patient was enrolled on a phase III trial. A skin lesion was noted when the first dose of natalizumab was administered, melanoma was diagnosed after five doses, and the patient died from melanoma (patient 1, Table 2) 21, 23. The number of doses of natalizumab before melanoma onset is of interest. Among the seven published cases and the index case, four individuals had received 1, 1, 5, and 12 natalizumab doses and four received large numbers of doses of natalizumab prior to melanoma diagnosis. A longer therapy duration would be expected if natalizumab caused melanoma via an immunologic pathway, unless existing nevi were already premalignant lesions. PML occurs after a long duration of rituximab and a short duration of brentuximab vedotin and ibrutinib (drugs used for hematologic malignancies), and after a variable time period following natalizumab 7, 8, 10, 13, 40, 41, 42, 43, 44, 46.

According to the National Cancer Institute's (NCI) Surveillance, Epidemiology and End Results (SEER) database, the median age of diagnosis for melanoma was 63 years 36. The index patient in this study was diagnosed at 43 years of age, the mean age of the 137 FAERS cases of natalizumab‐associated melanoma was 45 years, and the median age of the seven published reports was 41 years. The rare mucosal location of melanomas in our index case and several FAERs reported cases is notable. Only nine of the 137 (0.66%) melanoma patients with natalizumab exposure were reported as having died from melanoma. We note that in the FAERs database follow‐up to the end‐point of mortality may not be obtained consistently. Regardless, this mortality rate is less than that reported with de novo melanoma‐ based on data from SEER from 2006 to 2012, the 5‐year survival rate for all stages of melanoma is 91.5% 21. Uncommon locations of reported melanoma sites, younger median age of melanoma onset, and high survival rate among the eight cases of natalizumab‐associated melanoma reviewed herein raise questions about whether natalizumab‐associated melanoma is biologically different from de novo melanoma.

Multiple studies evaluated incidence of melanoma among MS patients, although none reported convincing epidemiologic or biologic associations or relative risk estimates 26, 27, 28, 37. Capkun et al. noted a slight decrease in melanoma rate among MS patients from a large sample, but do not report on melanoma risks among natalizumab‐treated MS patients 37. Another study reviewing incidence of cancer in MS (sampling 54,929 patients) concluded that melanoma rates were lower for people with MS compared to melanoma among the general population 26. One study reported on 74 patients with MS treated with natalizumab 38. They monitored 775 pigmented lesions and found no effect of natalizumab on proliferative and invasive phenotypes of melanoma cells in vitro 38.

**Figure 2:**
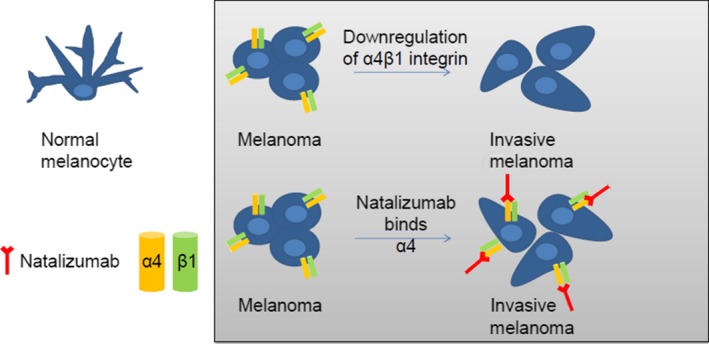
Proposed pathophysiology of natalizumab‐associated melanoma. Natalizumab promotes invasive melanoma by blocking α4 integrin. Normal melanocytes do not express α4β1 integrin; however, α4β1 integrin is expressed on melanoma cells. Downregulation of α4β1 integrin has been shown to be involved in melanoma invasion 32. Thus we hypothesize that natalizumab binding of α4 would promote melanoma through this mechanism.

Natalizumab is a humanized monoclonal antibody to the *α*4 component of the *α*4*β*1 integrin. Blocking *α*4 integrins on the surface of lymphocytes prevents their adhesion to vascular‐cell adhesion molecule 1 (VCAM‐1), which is expressed on the surface of vascular endothelial cells in brain and spinal cord blood vessels (the acknowledged mechanism of action for the favorable effect on MS). Studies of *α*4 integrin expression show that it has different roles in melanoma 30, 31, 32, 33. The underlying pathophysiology of a potential causal relationship is uncertain. (Fig. 2) Normal melanocytes and early stage melanomas do not express *α*4 integrin, whereas metastatic melanomas do 30, 31, 32, 33, 34, 35, 36. Expression of *α*4*β*1 integrin shows an inverse correlation with invasive potential of an aggressive melanoma cell line (B16) 30, 33. Expression of the integrin *α*4*β*1 on melanoma cells can inhibit the invasive stage of melanoma 34, 37. Thus, there is a concern that an antibody such as natalizumab that binds to *α*4 could directly promote at the cellular level melanoma cell replication, invasion, and migration.

The index case differs from previous case reports, and from 38% of FAERS cases, because there was no known mole or nevi that transformed upon natalizumab initiation, but rather a melanoma developed in a rare location unrelated to sun exposure. The initial informed consent form for natalizumab in clinical trials stated that its use could theoretically lead to increased risk of some types of cancers due to immune system effects. Three fatal cases of natalizumab‐associated PML were identified in clinical trials shortly after natalizumab received FDA approval in 2004. Natalizumab was temporarily withdrawn from the United States market in 2005 in response to concerns that immunodeficiency associated PML was not uncommon 29. In 2006, natalizumab was reintroduced in conjunction with the TOUCH safety registry that facilitates early PML identification and treatment.

Our study has safety implications. Overall FAERS reporting completeness was poor. The existence of the TOUCH Safety Surveillance Program, an FDA‐mandated program, did not improve melanoma reporting. Among 95 FAERS cases reported from the United States, only 15% were reported to FAERS from the TOUCH Safety Surveillance Program. While safety personnel at the manufacturer reviewed each adverse event report of natalizumab‐associated melanoma contained in FAERS, it is not easy to obtain follow‐up information on pathologic confirmation of melanoma outside of querying the TOUCH Safety Surveillance Program as was done for 88% of FAERS reports. Information on pathologic confirmation of melanoma was obtained for 38% of cases in the TOUCH Safety Surveillance Program database. As with all FAERS reports, complete follow‐up information, such as date of death, is often omitted. Median follow‐up for FAERS reports was 4 months, suggesting that long‐term follow‐up data may have been missing and hence long‐term survival rate may be underreported.

Various observational databases or registries with safety‐related events exist for natalizumab— most are manufacturer‐funded. These include the Tysabri Global Observational Program in Safety (TYGRIS), a study that contains information on 5000 patients. It examines incidence and pattern of serious infections, malignancies and other serious adverse events among natalizumab‐treated patients with multiple sclerosis in Canada and the United States. In an interim analysis based on follow‐up for 4821 natalizumab‐treated patients enrolled in a Tysabri Observational Protocol (TOP), an ongoing observational study conducted in Europe, Australia, Canada, and Argentina, 77% were followed up for at least 1 year and 52% for at least 2 years 46. The median follow‐up period was 26 months (range: 1–69). Eighteen patients were diagnosed with PML and one patient was diagnosed with melanoma 46. The distinction here is that while the Tysabri Safety Surveillance Program focuses on JCV‐associated PML and other opportunistic infections, TYGRIS, TOP, and other international registries focus on malignancies and opportunistic infections.

Some limitations can be identified. FDA case reporting is limited as only 1% of all adverse events, such as natalizumab‐associated melanoma, are included in FAERS and 25–50% completeness occurs in reporting of individual cases 6, 12, 45. Pathologic documentation was recorded in 78% of reports of melanoma cases reported to regulatory agencies versus 100% pathologic confirmation for the index case and the seven published cases. The manufacturer was able to review pathology for the three clinical trial participants who developed melanoma. Also, reporting of biopsies of nevi that did not contain melanoma was not included in many adverse event reports. For natalizumab‐associated melanoma, these limitations adversely impact causality and epidemiology investigations. The existence of the TOUCH Safety Surveillance Program did little to improve completeness of reporting, although it certainly increased the likelihood of physician reporting of natalizumab‐associated melanoma cases. Forty percent of natalizumab‐associated melanoma cases reported to regulatory agencies and 100% of cases of natalizumab‐associated melanoma reported in the literature were reported by physicians. This represents a markedly higher percentage reporting rate than that associated with adverse events reported with other drugs such as ticlopidine, clopidogrel, and warfarin (<5% reporting is from physicians) 45.

Future studies are needed. Comparison data from a population of melanoma patients who do or do not have MS should be evaluated, with a focus on characteristics of individuals with tumors in non‐sun‐exposed areas (including age at diagnosis, long‐term survival rates, and percent with prior histories of cutaneous nevi). Comprehensive long‐term follow‐up of persons with natalizumab‐associated melanoma should be obtained. Data on the incidence of melanoma in non‐sun‐exposed or noncutaneous areas would also be of interest.

We conclude that natalizumab‐treated individuals and their clinicians should be informed of potential risk of melanoma and be vigilant for changes in nevi appearance or development of new cutaneous lesions, particularly those that occur in non‐sun‐exposed cutaneous areas. MS patients with cutaneous nevi should consider photographing these lesions before natalizumab treatment is initiated. Recent reports on ocrelizumab, a new MS agent that depletes CD20‐expressing B cells, identified increased cancer occurrences in the treated arm compared to placebo arms (including one malignant melanoma case) 48, 49. Finally, the Tysabri Safety Surveillance Program should be expanded to include information on malignancies among natalizumab‐treated MS patients (Table 3). Increased risks of infection and neoplasms occur with all immunosuppressive treatments.

## Conflict of Interest

None declared.
